# Skeletal muscle high energy phosphate metabolism in patients with obesity and impaired fasting blood glucose

**DOI:** 10.1186/1532-429X-14-S1-P74

**Published:** 2012-02-01

**Authors:** Gurusher S Panjrath, Michael Schär, AbdEl-Monem El-Sharkawy, Steven P Schulman, Kerry Stewart, Gary Gerstenblith, Paul A Bottomley, Robert G Weiss

**Affiliations:** 1 Medicine/Cardiology, The Johns Hopkins University School of Medicine, Baltimore, MD, USA; 2 Radiology, The Johns Hopkins University School of Medicine, Baltimore, MD, USA

## Summary

To determine whether SM CK energy metabolism is impaired in obesity-associated IFG SM at rest and whether there is any relationship between SM CK energy metabolism and exercise capacity in these patients.

## Background

Skeletal muscle (SM) is the major site of glucose consumption and the creatine kinase (CK) reaction is the primary energy reserve in SM, providing additional ATP during ischemia and exercise. Although obesity-associated impaired fasting glucose (O+IFG) impairs SM glucose uptake and energy metabolism, it is not clear whether CK energy metabolism is also altered in O+IFG.

## Methods

Method: Ten obese participants with IFG (O+IFG), defined as BMI>30 and fasting glucose ≥100 mg/dl or HbA1c ≥ 5.7, twelve obese participants without IFG (O-IFG), and five non-obese subjects with normal fasting glucose underwent calf muscle 31P MRS with saturation transfer measures of CK metabolism at rest on a Philips 3T MRI/MRS system. The CK pseudo-first-order rate constant, k (sec-1) was calculated using TRiST and relative high-energy phosphate levels as previously described [1,2]. O-IFG and O+IFG subjects also underwent treadmill exercise testing for determination of maximal exercise time and peak oxygen consumption. Group comparison and correlations within each group were performed using XLSTAT package.

## Results

Results: Baseline demographics, anthropometric and metabolic measures, and 31P MRS results are detailed in Table 1. The mean CK pseudo-first order rate constant, k and mean PCr/ATP ratios were similar among all participant groups. There were no significant correlations between k and other exercise, metabolic or anthropometric parameters.

**Table 1:** 

	Healthy (n=5)	O-IFG (n=12)	O+IFG (n=10)
Age (yrs)	37.8 ± 8.7	52.4 ± 8.1*	52.8 ± 7.7*
Male %	100	25	20
BMI	24.0 ± 3.0	35.4 ± 5.1*	34.5 ± 4.4*
Plasma glucose (mg/dl)	-	90.8 ± 5.6	114.4 ±14.2¥
HbA1c	-	5.2 ± 0.3	5.6 ± 0.5§
Metabolic syndrome %	-	50	40
VO2 ml/kg/min	-	25.7 ± 4.4	24.2 ± 4.3
ETT(min)		914.7 ± 288	809.6 ± 232
PCr/ATP	6.27 ± 0.52	6.52 ± 0.59	6.85 ± 0.49
k (s-1)	0.29 ± 0.03	0.28 ± 0.03	0.26 ± 0.04

Mean±standard deviation. BMI- body mass index; HbA1c- Glycosylated hemoglobin; VO2- peak oxygen consumption; ETT- Treadmill Exercise Time; PCr/ATP- phosphocreatine/adenosine triphosphate ratio; k- CK reaction pseudo-first order rate constant. * p< 0.01 compared to healthy; § p < 0.05 and ¥ p<0.001 compared to O-IFG.

## Conclusions

We believe this is the first report on CK kinetics in resting SM in obese subjects with impaired fasting glucose. The findings indicate that intracellular ATP CK kinetics are preserved at rest in these subjects compared with obese subjects with normal glucose or healthy subjects. ATP kinetics via CK of resting SM do not appear to be associated with indices of glycemic control or exercise capacity. Thus, SM CK high-energy phosphate levels and ATP kinetics appear to be normal at rest in obese subjects with and without IFG. The response of CK metabolism during exercise in obese subjects is yet to be determined.

## Funding

NIH HL61912 and Clarence Doodeman Endowment.
